# Targeted antigen delivery to dendritic cells elicits robust antiviral T cell-mediated immunity in the liver

**DOI:** 10.1038/srep43985

**Published:** 2017-03-07

**Authors:** Julia Volckmar, Marcus Gereke, Thomas Ebensen, Peggy Riese, Lars Philipsen, Stefan Lienenklaus, Dirk Wohlleber, Robert Klopfleisch, Sabine Stegemann-Koniszewski, Andreas J. Müller, Achim D. Gruber, Percy Knolle, Carlos A. Guzman, Dunja Bruder

**Affiliations:** 1 Immune Regulation Group, Helmholtz Centre for Infection Research, Braunschweig, Germany & Infection Immunology Group, Institute of Medical Microbiology, Infection Control and Prevention, Medical Faculty of the Otto-von-Guericke University Magdeburg, Magdeburg, Germany; 2 Department of Vaccinology and Applied Microbiology, Helmholtz Centre for Infection Research, Braunschweig, Germany; 3 Intravital Microscopy in Infection and Immunity, Institute for Molecular and Clinical Immunology, Medical Faculty of the Otto-von-Guericke University Magdeburg, Magdeburg, Germany; 4 Department of Molecular Immunology, Helmholtz Centre for Infection Research, Braunschweig, Germany; 5 Institute of Molecular Immunology, Technische Universität München, Germany; 6 Department of Veterinary Medicine, Institute of Veterinary Pathology, Free University Berlin, Berlin, Germany; 7 Institute of Molecular Medicine and Experimental Immunology, Universität Bonn, Germany

## Abstract

Hepatotropic viruses such as hepatitis C virus cause life-threatening chronic liver infections in millions of people worldwide. Targeted *in vivo* antigen-delivery to cross-presenting dendritic cells (DCs) has proven to be extraordinarily efficient in stimulating antigen-specific T cell responses. To determine whether this approach would as well be suitable to induce local antiviral effector T cells in the liver we compared different vaccine formulations based on either the targeting of DEC-205 or TLR2/6 on cross-presenting DCs or formulations not involving *in vivo* DC targeting. As read-outs we used *in vivo* hepatotropic adenovirus challenge, histology and automated multidimensional fluorescence microscopy (MELC). We show that targeted *in vivo* antigen delivery to cross-presenting DCs is highly effective in inducing antiviral CTLs capable of eliminating virus-infected hepatocytes, while control vaccine formulation not involving DC targeting failed to induce immunity against hepatotropic virus. Moreover, we observed distinct patterns of CD8^+^ T cell interaction with virus-infected and apoptotic hepatocytes in the two DC-targeting groups suggesting that the different vaccine formulations may stimulate distinct types of effector functions. Our findings represent an important step toward the future development of vaccines against hepatotropic viruses and the treatment of patients with hepatic virus infection after liver transplantation to avoid reinfection.

The liver is permanently exposed to a plethora of antigens and microbial products with potentially immune-stimulatory capacity. The predominantly tolerogenic microenvironment of the liver usually prevents the induction of immunity to these innocuous antigens while at the same time it favours the establishment of persistent liver infection^1^,^2^. Next to other hepatotropic viruses, such as cytomegalovirus (CMV) or hepatitis B virus (HBV), a clinically highly relevant example for pathogens capable of establishing life-threatening chronic infections in the liver is the hepatitis C virus (HCV)^3^. Despite extensive research since the discovery of HCV in 1989^4^, an effective vaccine is still not available^5^.

Dendritic cells (DCs) represent optimal targets for designing effective vaccines^6^. CD8α^+^ DCs are unique with respect to their capacity to effectively cross-present exogenous antigens on MHC-I molecules to induce cytotoxic T cells (CTLs) in addition to Th1 responses^7^,^8^. Accordingly, CD8α^+^ DCs play a key role in establishing antiviral immunity^9^,^10^. Increasing knowledge regarding the characteristics of pattern recognition receptor (PRR) expression by different DC subsets has set the basis for a directed targeting of antigen *in vivo* by means of ligands or antibodies specific for the respective PRRs expressed on DCs. In this context, particularly Toll-like receptors (TLRs) and C-type lectin receptors (CLRs) gained importance^11^. For instance, the TLR2/6 heterodimer agonist S-[2,3-bispalmitoyloxy-(2R)-propyl]-R-cysteinyl-amido-mono-methoxyl polyethylene glycol (BPPcysMPEG), a synthetic derivative of the macrophage-activating lipopeptide (MALP-2), effectively targets cross-presenting CD8α^+^ DCs. Importantly, co-administration of BPPcysMPEG together with soluble ovalbumin (OVA) (OVA + BPPcysMPEG) resulted in the induction of OVA-specific CTLs^12^. Interestingly, BPPcysOVAMPEG, a compound consisting of the immunodominant OVA peptides chemically linked to BPPcysMPEG and therefore specifically delivered to TLR2/6 positive DCs, was even more effective at inducing OVA-specific CTLs^12^.

Next to the TLR2/6 heterodimer, CD8α^+^ DCs express high levels of the CLR family endocytosis receptor DEC-205^13^. Importantly, receptor-mediated antigen uptake by CD8α^+^ DCs via DEC-205 results in extraordinarily effective antigen cross-presentation to CD8^+^ T cells^14^,^15^,^16^,^17^,^18^. Steinman and colleagues demonstrated that *in vivo* targeting of antigen to cross-presenting DCs by means of DEC-205-directed antibody-antigen conjugates together with the appropriate adjuvants resulted in a potent induction of specific T cell responses^19^,^20^. Follow up studies with viral^14^,^16^,^17^,^21^, bacterial^22^,^23^ and tumour antigens^24^,^25^ proved DEC-205-mediated antigen delivery to CD8α^+^ DCs to elicit protective CD4^+^ and CD8^+^ T effector cells. However, no study so far addressed whether antigen delivery to cross-presenting CD8α^+^ DCs is able to induce effector T cell responses and antiviral immunity in the liver. To improve vaccination efficacy against hepatotropic viruses, we compared different vaccine formulations regarding their potency to induce antiviral effector T cell responses in the liver. This included targeted antigen delivery to cross-presenting DCs by αDEC-205 conjugated to the OVA protein (αDEC-205/OVA adjuvanted with Poly(I:C)/CpG) and the less well studied BPPcysOVAMPEG containing the two immunodominant MHC-I and -II OVA peptides. To assess whether antigen targeting to DCs would be required for inducing antiviral effector T cells in the liver, another group that received OVA co-administered with BPPcysMPEG (OVA + BPPcysMPEG) and thus not involving targeted antigen delivery to DCs was included. We show that only immunization with the DC targeting formulation αDEC-205/OVA and BPPcysOVAMPEG but not OVA + BPPcysMPEG vaccination induced CD8^+^ effector T cells capable of eliminating virus infected hepatocytes. Thus, we conclude that targeted *in vivo* antigen delivery to cross-presenting DCs represents a promising approach for the induction of antiviral immunity in the liver with potential implications for the development of vaccines against hepatotropic viruses.

## Results

### Targeting antigen to DCs induces humoral immunity

We first compared the OVA-specific humoral immune response after immunization with either αDEC-205/OVA adjuvanted with Poly(I:C) and CpG (αDEC-205/OVA + Poly(I:C)/CpG; for simplification termed αDEC-205/OVA), BPPcysOVAMPEG or, in addition to the two DC targeting approaches, BPPcysMPEG co-administered together with soluble OVA (OVA + BPPcysMPEG). As controls we included αDEC-205 and OVA alone, both adjuvanted with Poly(I:C) and CpG as well as BPPcysMPEG alone, OVA peptides alone, and OVA peptides adjuvanted with BPPcysMPEG.

**Figure 1:**
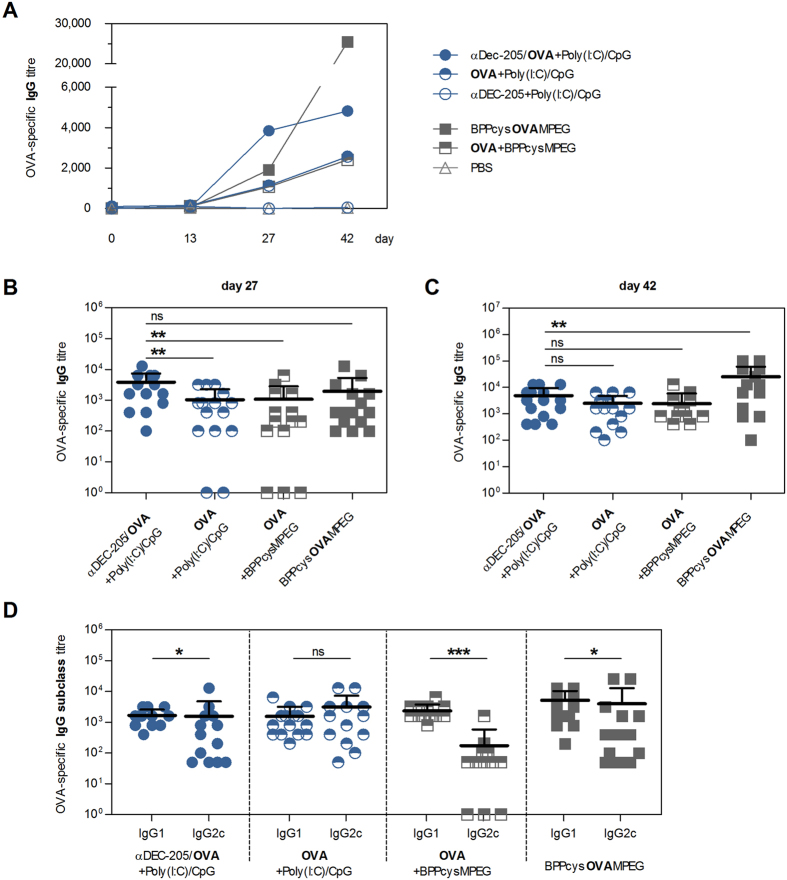
Antigen targeting to DCs induces IgG responses of distinct kinetics and subtype distribution. Mice (n = 5) were immunized on days 0, 14 and 28 with αDEC-205/OVA + Poly(I:C)/CpG, αDEC-205 + Poly(I:C)/CpG, BPPcysOVAMPEG, OVA + Poly(I:C)/CpG, OVA + BPPcysMPEG or PBS followed by determining OVA-specific serum IgG titres. Results are compiled from three independent experiments. (**A**) Kinetic of OVA-specific serum IgG titres. (**B**) OVA-specific IgG titre on day 27 and (**C**) day 42. Statistics: one-way Anova (mean ± SD) (**p < 0.01). (**D**) OVA-specific serum IgG_1_ and IgG_2c_ titres on day 42. Statistics: non-parametric Mann Whitney test comparing the IgG_1_ to IgG_2c_ subclasses within the groups (mean ± SD) (*p ≤ 0.015, ***p < 0.0001).

Already after two vaccinations with αDEC-205/OVA, we observed a strong OVA-specific IgG response (Fig. 1A) that was significantly increased in comparison to both the OVA + BPPcysMPEG and the OVA + Poly(I:C)/CpG groups at this time (Fig. 1B). Mice vaccinated with the novel antigen targeting system BPPcysOVAMPEG initially exhibited a weaker IgG response, which was however strongly enhanced following the third boost (Fig. 1A,C).

We next compared OVA-specific IgG_1_ and IgG_2c_ titres which represent the main IgG isotypes stimulated by Th2 and Th1 cells, respectively^26^. Vaccination with either αDEC-205/OVA, OVA + Poly(I:C)/CpG or BPPcysOVAMPEG induced a rather balanced IgG_1_/IgG_2c_ ratio, whereas OVA + BPPcysMPEG induced substantially higher IgG_1_ than IgG_2c_ titres, pointing towards a more Th2-dominated immune response (Fig. 1D). As expected, neither the injection of the adjuvant BPPcysMPEG alone, nor the vaccination with the immunodominant CD4^+^ and CD8^+^ OVA peptides without adjuvant did induce humoral immunity. Moreover, peptide targeting to DCs by means of vaccination with BPPcysOVAMPEG was superior in inducing antibody responses than vaccination with soluble peptides adjuvanted with BPPcysMPEG (Supplementary Fig. S1A–C).

### Induction of CTLs following αDEC-205/OVA and BPPcysOVAMPEG immunization

**Figure 2:**
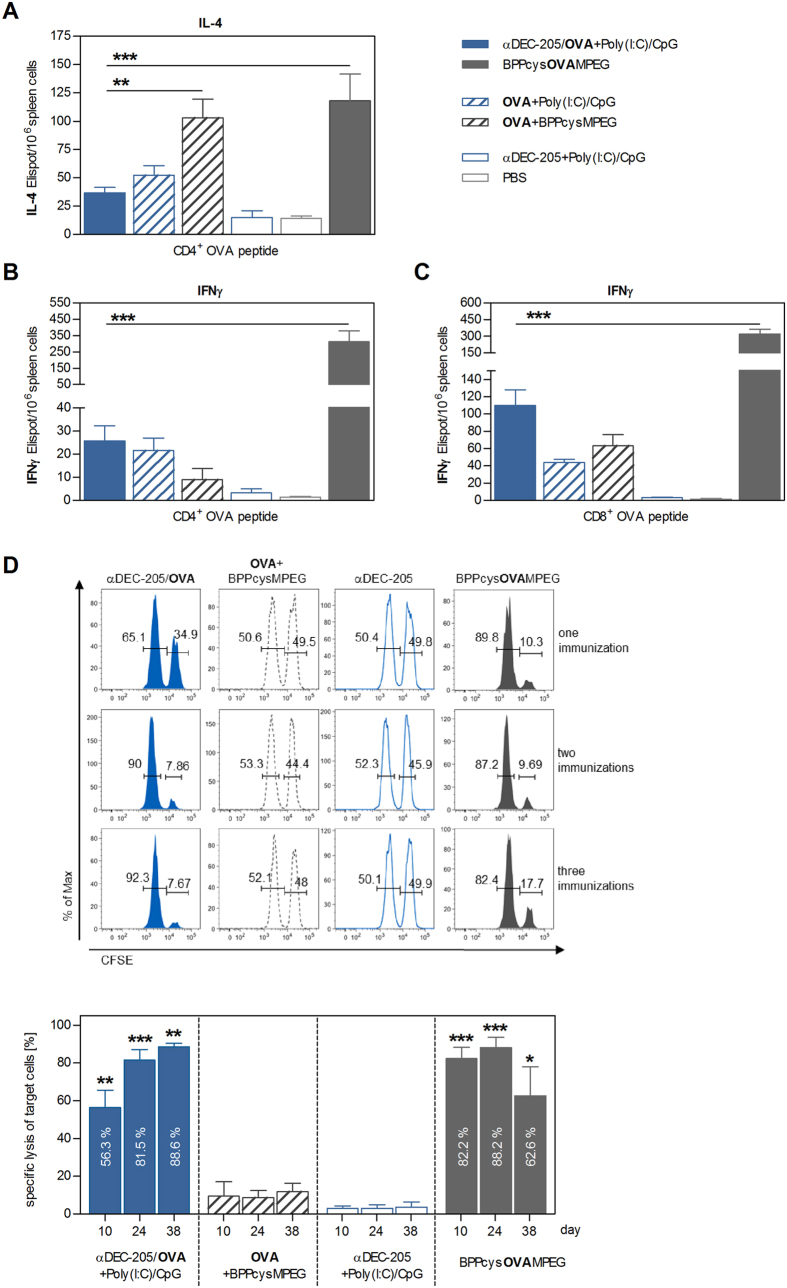
Induction of IFNγ-producing CTLs following αDEC-205/OVA and BPPcysOVAMPEG immunization. Mice were immunized days 0, 14 and 28 with αDEC-205/OVA + Poly(I:C)/CpG, αDEC-205 + Poly(I:C)/CpG, BPPcysOVAMPEG, OVA + Poly(I:C)/CpG, OVA + BPPcysMPEG or PBS. IL-4 (**A**) or IFNγ (**B**), (**C**) spot forming units/10^6^ splenocytes following stimulation with the indicated OVA-peptides on day 42. Bars represent the mean ± SEM (n = 5, triplicates from pooled animals) of three independent experiments (**p < 0.01, ***p < 0.0001). (**D**) *In vivo* cytotoxicity assay: mice (n = 3) were immunized once, twice or thrice. Histograms: CFSE^+^ splenocytes in one representative mouse per group and per point in time. Bars: percentage of specific lysis as mean ± SEM. Statistics: one-way Anova (*p < 0.05, **p < 0.01, ***p ≤ 0.0007).

To further characterize the type of T cell response induced with the different vaccine formulations, OVA-specific IL-4- and IFNγ-secreting T cells were evaluated by *ex vivo* stimulation of splenocytes from immunized mice with the dominant OVA MHC-I and -II peptides. As expected, due to the exclusive selection towards these epitopes when immunizing with BPPcysOVAMPEG, the highest number of cytokine producing T cells was observed in this group (Fig. 2A–C). BPPcysOVAMPEG vaccination induced increased numbers of IL-4-producing Th2 cells compared to the αDEC-205/OVA and OVA + Poly(I:C)/CpG immunized groups (Fig. 2A). Compared to the vaccine formulations lacking direct antigen targeting to DCs, BPPcysOVAMPEG, and to a lesser extent αDEC-205/OVA immunization, at the same time showed the strongest potential to induce IFNγ-producing T cells (Fig. 2B,C). Of note, the adjuvant BPPcysMPEG alone or vaccination with non-adjuvanted immunodominant OVA peptides did not induce IFNγ-producing T cells. Furthermore, peptide targeting to DCs using the BPPcyOVAMPEG formulation induced significantly more IFNγ-producing T cells than vaccination of mice using peptides adjuvanted with BPPcysMPEG (Supplementary Fig. S1D–G).

Since IFNγ-producing CTLs are important for virus clearance from the liver^27^, we evaluated OVA-specific CTL activity in vaccinated mice. Immunization with αDEC-205/OVA or BPPcysOVAMPEG induced a robust OVA-specific CTL response already after the first immunization (Fig. 2D). Despite the fact that IFNγ-producing CD8^+^ T cells were also induced in OVA + BPPcysMPEG immunized mice (Fig. 2C) no lysis of target cells was detectable in this group (Fig. 2D). Thus, while antigen delivery to DCs by targeting DEC-205 resulted in a rather weak and balanced Th1 (IFNγ) and Th2 (IL-4) cell response, it was highly effective in generating antigen-specific CTLs. Similarly, BPPcysOVAMPEG-mediated antigen targeting to TLR2/6 induced balanced Th1/Th2 T helper and robust OVA-specific CTL responses.

### Targeted antigen delivery to DCs induces IFNγ-producing memory CD8^+^ T cells in the liver

**Figure 3:**
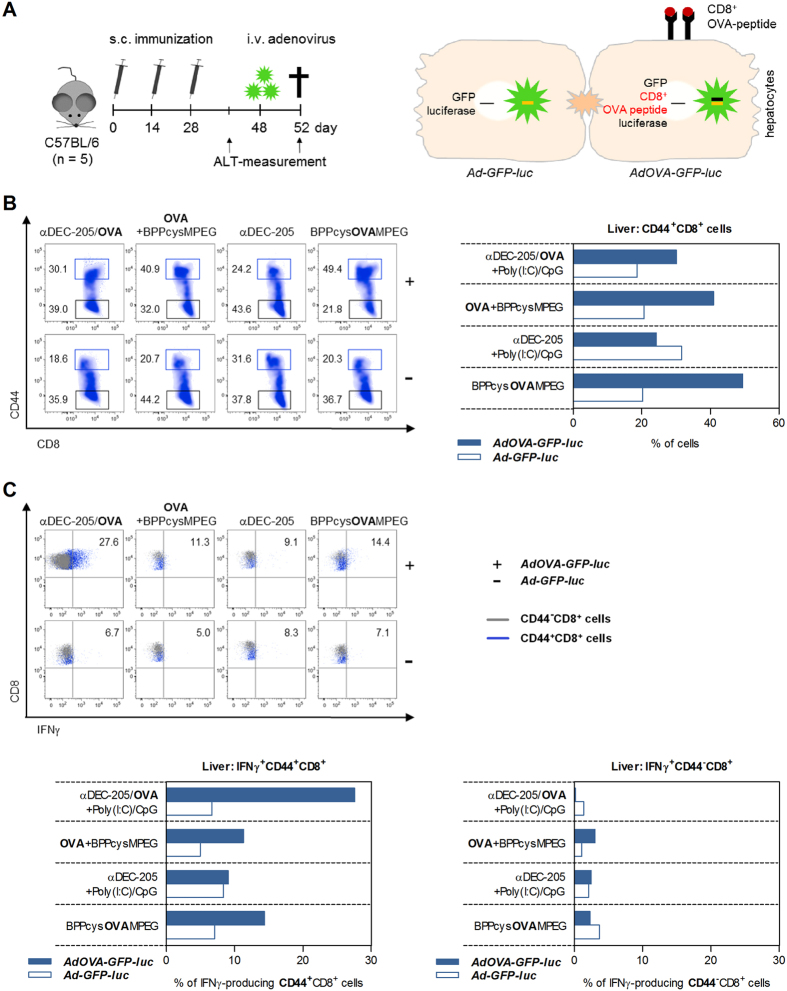
Antigen targeting to DCs induces accumulation of IFNγ^+^CD8^+^ T cells in the liver. (**A**) Experimental setup: adenovirus challenge assay. Mice (n = 5) were immunized on days 0, 14 and 28 either with αDEC-205/OVA + Poly(I:C)/CpG, αDEC-205 + Poly(I:C)/CpG, BPPcysOVAMPEG or OVA + BPPcysMPEG and challenged with *AdOVA-GFP-luc* or *Ad-GFP-luc* on day 48. (**B**) 4 days after infection, liver lymphocytes were analysed by flow cytometry. Representative density plots show CD8^+^ liver T lymphocytes with the percentages of CD44^+^ and CD44^−^ expression. Bars represent the proportions of CD44^high^CD8^+^ cells. (**C**) Overlays show IFNγ-producing CD44^−^CD8^+^ (grey) and CD44^high^CD8^+^ (blue) liver T cells. Bars display the percentages of IFNγ-producing CD44^−^CD8^+^ (left) and CD44^high^CD8^+^ (right) cells.

To test whether CTLs induced by antigen delivery to DCs can effectively recognize and eliminate virus infected hepatocytes, a hepatotropic adenovirus challenge model was utilized (Fig. 3A). Immunized mice were infected with a recombinant adenovirus (*AdOVA-GFP-luc*) leading to the MHC-I presentation of the OVA CD8_257–264_ peptide on infected hepatocytes. Furthermore, *AdOVA-GFP-luc* infected cells express EGFP (enhanced green fluorescent protein) and luciferase, allowing the quantification of the infection (Fig. 3A)^28^. While infection of αDEC-205 mock-immunized mice with *AdOVA-GFP-luc* did not lead to an accumulation of CD44^high^CD8^+^ T cells in the liver, an accumulation of intrahepatic CD44^high^CD8^+^ T cells was detectable in all other experimental groups following *AdOVA-GFP-luc* infection (Fig. 3B).

The release of IFNγ by CTLs has been shown to mediate non-cytolytic clearance of infected hepatocytes and IFNγ *per se* directly inhibits viral replication in the liver^29^,^30^. While hardly any IFNγ production was detectable in the hepatic CD44^-^CD8^+^ T cells (Fig. 3C), the *AdOVA-GFP-luc* challenge resulted in a nearly two-fold increase of the proportion of IFNγ-producing CD44^high^CD8^+^ T cells in the OVA + BPPcysMPEG (11.3%) and BPPcysOVAMPEG (14.4%) immunized animals. Strikingly, αDEC-205/OVA vaccination resulted in an exceptionally strong accumulation of IFNγ-producing CD44^high^CD8^+^ effector memory T cells (27.6%) in the liver following *AdOVA-GFP-luc* infection (Fig. 3C).

### Presence of IFNγ^+^CD44^high^CD8^+^ effector T cells in the liver correlates with hepatitis development following *AdOVA-GFP-luc* challenge

**Figure 4:**
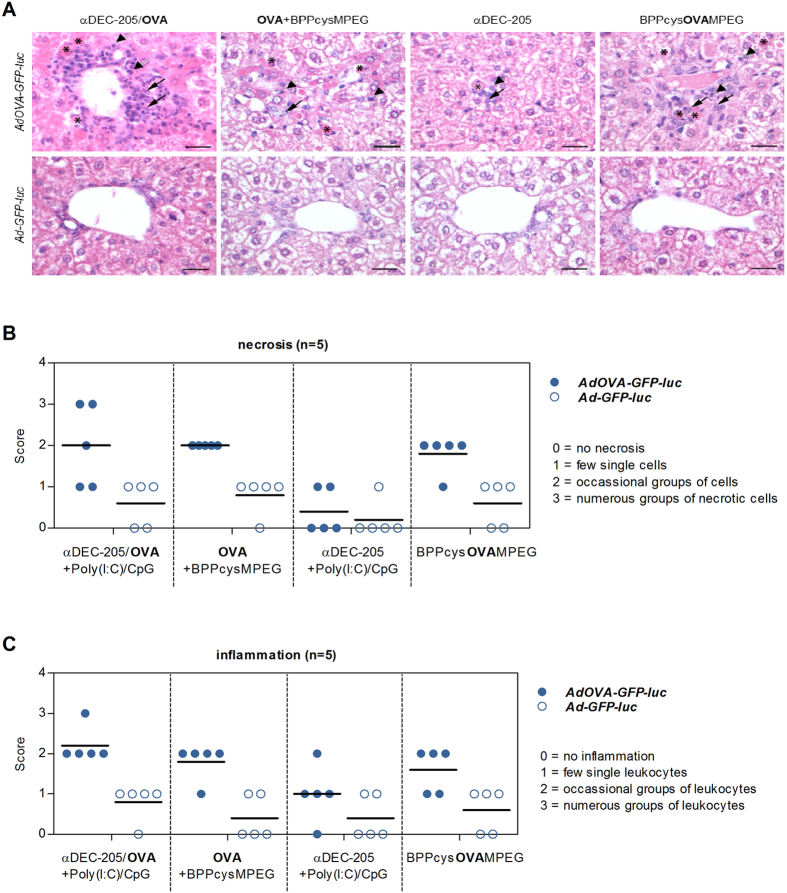
Adenovirus challenge induces antigen-specific liver pathology. Mice (n = 3) were immunized on days 0, 14 and 28 either with αDEC-205/OVA + Poly(I:C)/CpG, αDEC-205 + Poly(I:C)/CpG, BPPcysOVAMPEG or OVA + BPPcysMPEG and three weeks after the last immunization they were infected with *AdOVA-GFP-luc* or *Ad-GFP-luc*. On day 4 after adenovirus infection, livers were harvested for histological examination. (**A**) Macrophages (arrow), lymphocytes (arrowhead) and necrotic hepatocytes (*) are indicated (scale bar = 100 μm). (**B**) Necrosis and (**C**) tissue inflammation was assessed. 5 mice per group were analysed. Mean of scattered dot plots are represented.

**Figure 5:**
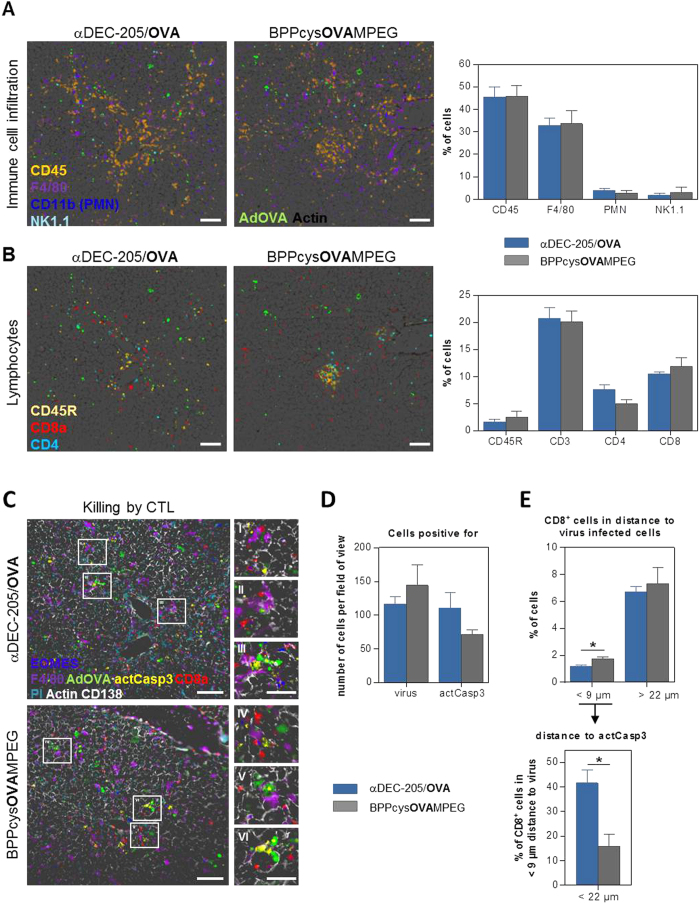
Distinct CD8^+^ T cell/hepatocyte interaction pattern in virus infected livers of αDEC-205/OVA and BPPcysOVAMPEG immunized mice. Mice (n = 3–4) were immunized on days 0, 14 and 28 either with αDEC-205/OVA + Poly(I:C)/CpG or BPPcysOVAMPEG and challenged with *AdOVA-GFP-luc* or *Ad-GFP-luc* control. 4 days after adenovirus infection, liver lobes were harvested for MELC analysis. (**A**,**B**) Tissue sections of immunized and *AdOVA-GFP-luc* infected mice. Scale bars = 100 μm. Graph: quantification of immune cell subsets as percentages of all detected cells. (**A**) Immune cell infiltration: black = actin, liver structure; green = GFP-tagged virus (*AdOVA-GFP-luc)*; orange = leukocytes (CD45); purple = macrophages (F4/80); blue = neutrophils (PMN) (CD11b^high^); cyan = natural killer cells (NK1.1). (**B**) Lymphocytes: light yellow = B cells (CD45R^+^); red = CTL (CD8); light blue = CD4 T cell. (**C**) Interaction of virus infected cells with CTLs: green = GFP-tagged virus (*AdOVA-GFP-luc*); yellow = apoptotic cell (active caspase-3^+^); red = CTL (CD8); white = liver structure (actin + CD138); blue/green = nuclei (propidiumiodid); blue = eomes; purple = macrophages (F4/80). Scale bars = 100 μm or 50 μm (zoom). (**D**) Quantification of virus infected cells or active caspase-3^+^ cells: n = 3 with 2–4 fields of view were measured per animal. (**E**) Localization analysis of CD8^+^ T cells in relation to virus infected cells and apoptotic cells. Upper graph: percentages of CD8^+^ cells in direct (<9 μm) or loose contact (>22 μm) to virus infected cells. Lower graph: percentages of CD8^+^ cells in direct contact (<9 μm) to virus infected cells which are close (<22 μm) to cells expressing active caspapse-3. Statistics: paired, two-tailed *t*-test (*p < 0.05).

We next asked whether the IFNγ-producing effector memory CD8^+^ T cells in the liver would respond to virus-expressed antigen on hepatocytes. While *Ad-GFP-luc* control infection induced only marginal hepatocellular cell death or cellular infiltrations (Fig. 4A), infection of all OVA-immunized groups with *AdOVA-GFP-luc* led to drastic histological lesions in the liver, hepatocellular cell death and marked infiltration of lymphocytes and macrophages into the tissue (Fig. 4A–C). Since histological scoring revealed a trend towards an enhanced immunopathology in the liver of αDEC-205/OVA immunized and *AdOVA-GFP-luc* infected mice compared to the BPPcysOVAMPEG group, we further elucidated whether αDEC-205/OVA and BPPcysOVAMPEG vaccination would result in distinct immune cell recruitment following viral infection. However, automated multidimensional fluorescent microscopy (MELC) on liver sections revealed no significant differences in the hepatic composition of leukocytes, macrophages, neutrophils, NK cells, B cells and T cell subsets among the groups (Fig. 5A,B and Supplementary Fig. S2). Altogether, the observed type of immunopathology in αDEC-205/OVA and BPPcysOVAMPEG immunized mice was indicative for acute hepatitis as a consequence of the immune-mediated clearance of virus infected hepatocytes suggesting that antigen-specific CTLs present in the liver following vaccination accounted for the hepatocyte damage.

### Vaccination with αDEC-205/OVA and BPPcysOVAMPEG results in effective antigen-specific virus clearance in the liver

**Figure 6:**
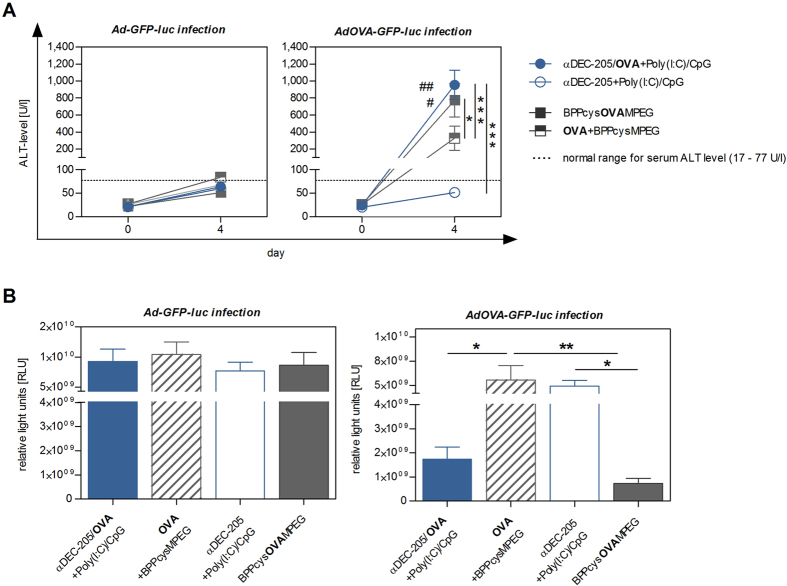
Antigen delivery to DCs induces CTLs that clear virus infected hepatocytes. Mice were immunized on days 0, 14 and 28 either with αDEC-205/OVA + Poly(I:C)/CpG, αDEC-205 + Poly(I:C)/CpG, BPPcysOVAMPEG or OVA + BPPcysMPEG and infected with *AdOVA-GFP-luc* or *Ad-GFP-luc* on day 48. (**A**) Quantification of ALT levels before and after adenovirus challenge. Data represent the mean values (±SEM) (n = 5). Statistics: two-way RM Anova (*p < 0.05; ***p < 0.001) or paired, two-tailed *t*-test comparing values from day 0 and 4 within the respective group (^#^p = 0.019; ^##^p = 0.0056). (**B**) Luciferase activity in the liver as a measure for killing of infected hepatocytes. Relative light units expressed as mean ± SEM (n = 5). Statistics: one-way Anova (*p < 0.05; **p ≤ 0.0014).

To further substantiate the cytolytic function of CTLs, serum concentrations of alanine transaminase (ALT) were determined in vaccinated and adenovirus-challenged animals. As expected, infection with the *Ad-GFP-luc* control virus did not affect serum ALT levels in any of the experimental groups (Fig. 6A). Strikingly, in αDEC-205/OVA as well as in BPPcysOVAMPEG immunized mice we observed a dramatic and comparably high increase of the serum ALT level (~950 U/l and ~770 U/I, respectively) indicating massive CTL-mediated killing of *AdOVA-GFP-luc* infected hepatocytes (Fig. 6A). Significantly lower ALT concentrations (~330 U/l) were measured in OVA + BPPcysMPEG immunized mice which is in line with lower abundance of IFNγ-producing memory CD8^+^ T cells (Fig. 3).

To further confirm specific killing of *AdOVA-GFP-luc* infected hepatocytes, virus clearance was examined by assessing luciferase expression in liver tissue. As expected, strong luciferase activity was detectable in the liver of all mice infected with the control *Ad-GFP-luc* virus (Fig. 6B). In contrast, luciferase activity in the livers of immunized and *AdOVA-GFP-luc* infected mice was significantly reduced in αDEC-205/OVA and BPPcysOVAMPEG vaccinated mice in comparison to both OVA + BPPcysMPEG and αDEC-205 + Poly(I:C)/CpG mock-immunized animals (Fig. 6B). Taken together, our results clearly demonstrated that antigen targeting to cross-presenting DCs by either αDEC-205/OVA or BPPcysOVAMPEG is highly effective at inducing effector T cells capable of recognizing and eliminating virus infected hepatocytes.

### Vaccination with αDEC-205/OVA and BPPcysOVAMPEG induces distinct patterns of CD8^+^ T cell interaction with virus infected and apoptotic hepatocytes

In order to gain first indication regarding the underlying mechanisms for the elimination of infected hepatocytes we analysed liver sections of αDEC-205/OVA and BPPcysOVAMPEG immunized and *AdOVA-GFP-luc* infected mice for potential differences in the distance of CD8^+^ T cells to virus infected hepatocytes. While we did neither observe significant differences in the number of virus infected hepatocytes, nor in the number of hepatocytes staining positive for active caspase-3 (Fig. 5C,D) or in the frequency of CD8^+^ T cells being in loose contact with infected hepatocytes (>22 μm distance, Fig. 5E), we indeed observed significantly fewer CD8^+^ T cells being in direct contact (<9 μm) to *AdOVA-GFP-luc* infected hepatocytes in αDEC-205/OVA immunized mice compared to the BPPcysOVAMPEG immunized group (Fig. 5E). Strikingly, despite lower abundance of CD8^+^ T cells being in intimate contact with virus infected cells, substantially more of them were found to be closely associated with active caspase-3 and thus apoptotic cells in the liver of αDEC-205/OVA immunized mice (Fig. 5E). Together with the observation that compared to BPPcysOVAMPEG vaccination αDEC-205/OVA immunization resulted in the accumulation of significantly higher frequency of IFNγ^+^CD44^high^CD8^+^ effector T cells in virus infected livers (Fig. 3C) these data may indicate that the two different DC-targeting formulations may indeed stimulate distinct types of effector functions being active during virus elimination from the liver.

## Discussion

We show here that *in vivo* antigen delivery to CD8α^+^ cross-presenting DCs is highly effective in inducing antiviral immunity in the liver. BPPcysOVAMPEG vaccination, which was included as a novel antigen targeting system, exhibited outstanding properties in inducing high antibody titres (Fig. 1), Th1 and Th2 cells, as well as CTLs (Fig. 2) that effectively cleared virus infected hepatocytes (Fig. 6). Although not reaching statistical significance compared to αDEC-205/OVA vaccination, this approach tended to promote a more robust viral clearance, as well as slightly less pronounced hepatocellular cell death and pro-inflammatory effect. The value of TLR agonist-antigen conjugates for inducing Th1 and CD8^+^ T cell responses are clearly underscored by our new data and previous studies^31^,^32^. However, for clinical application such conjugates will need to be tailored to match different HLA alleles and rapidly mutating viruses (e.g. HCV)^33^. It could be possible to exploit chemical groups of BPPcysMPEG to generate conjugates with more complex antigens or to engineer universal docking systems enabling non-covalent binding, such as those used in the past for antibodies^34^.

Prajeeth *et al*. have shown that BPPcysMPEG-mediated activation of CD8^+^ DCs led to effective cross-priming against co-administered antigen resulting in CTL responses in the spleen^12^. While we detected small numbers of CD44^+^CD8^+^ memory T cells in the liver of OVA + BPPcysMPEG immunized mice following *AdOVA-GFP-luc* infection (Fig. 3), these cells did not exert effector functions (Figs 2 and 6). These discrepancies may be due to differences in the antigen concentration used (3 mg vs. 7 μg)^12^. However, we have no explanation yet why the memory T cells present in the liver of OVA + BPPcysMPEG immunized mice did not exhibit cytotoxic function. Strikingly, despite the lack of viral eradication in the OVA + BPPcysMPEG vaccinated group, these mice displayed marked immunopathology and elevated ALT levels following *AdOVA-GFP-luc* infection. We may speculate that, although being functionally impaired in terms of cytotoxicity, antigen-specific recognition of the virus by IFNγ^+^CD44^+^CD8^+^ T cells results in cytokine release followed by innate immune activation and immune cell recruitment that finally result in hepatic cell death and inflammation independent of CTL mediated killing.

Immunization with αDEC-205/OVA induced a robust CTL response (Fig. 2), which resulted in effective viral clearance in the liver (Fig. 6). Our data are well in line with a previous study demonstrating that vaccination with αDEC-205/OVA induces CD8^+^ T cells capable to protect against tumour or virus challenge^14^. Similar results were obtained by targeting tumour^24^,^25^ or viral antigens^21^,^35^ to DEC-205^+^ DCs. Next to the CTL response induced in αDEC-205/OVA immunized mice, we detected antigen-specific CD4^+^ T cells. While previous studies described a preferential induction of Th1 responses by αDEC-205/antigen immunization^16^,^23^, we observed similar levels of Th1 (IFNγ^+^) and Th2 (IL-4^+^) CD4^+^ T cells (Fig. 2A and B), consistent with minor differences in the IgG subclasses (Fig. 1C). Irrespectively of the antibody subclasses, we demonstrate that in comparison to the other formulations the two DC targeting approaches were most effective at inducing a rapid and robust humoral immune response (Fig. 1).

While in our hands both DC targeting approaches next to CTL responses induced a rather balanced Th1/Th2 profile, immunization with OVA + BPPcysMPEG that does not involve DC targeting failed to induce CTLs and resulted in a Th2 dominated cytokine profile (Figs 1C and 2). Interestingly, BPPcysMPEG has been shown to induce Th1-type immune responses in the context of allergens and parasite antigens^36^,^37^,^38^. However, intramuscular immunization with hepatitis B surface antigen virus-like particles co-administered with BPPcysMPEG resulted in a Th2 dominated response^39^. Therefore, the adjuvant dose, the route of administration and the intrinsic features of the antigen may account for the observed differences in the T helper cell phenotype. While Schulze and colleagues proved BPPcysMPEG to be a potent mucosal adjuvant for vaccination against severe acute respiratory syndrome coronavirus, the authors focused on the cellular and humoral immune responses, but did not test for antiviral activity^40^. Thus, BPPcysMPEG has not been investigated before regarding its potency to induce antiviral immunity. In the absence of DC targeting, i.e. by using the OVA + BPPcysMPEG formulation, we clearly show the induction of a Th2-biased CD4^+^ T cell phenotype, the lack of CTLs and in consequence no antigen-specific clearance of virus infected hepatocytes. While previous studies have demonstrated the exceptional potential of DEC-205-mediated antigen targeting to elicit adaptive immunity^14^,^15^,^16^,^17^,^18^,^24^ to our knowledge this is the first study showing the induction of liver-specific immune reactions. We demonstrate that targeting DEC-205^+^ DCs resulted in a high frequency of local IFNγ^+^CD44^+^CD8^+^ memory T cells (Fig. 3C), which correlate with protection against adenovirus challenge in the liver (Fig. 6). One critical point in fighting viral infections is to keep the balance between protective immunity and immunopathology, which are both mainly driven by CTLs and often decisive for the fate of the infected host^41^,^42^. For hepatotropic viruses such as HCV, which is not believed to be directly cytopathic, liver damage is attributed to T cell-mediated immunity^29^ and immunopathology is inevitable for sterilizing immunity. On the other hand, a less vigorous CTL response allows for viral persistence, ultimately leading to progressive tissue injury^43^. Only in the αDEC-205/OVA and BPPcysOVAMPEG group could immunopathology be correlated with antigen-specific viral clearance in the liver (Fig. 6). Regarding the severity of hepatitis that occurs as a side effect of pathogen elimination, it is highly conceivable that αDEC-205/OVA and BPPcysOVAMPEG immunized mice would have recovered from liver damage. In a published model of fulminant hepatic failure, ALT levels peaked at day 4–5 post infection, which was the end-point of the experiments in our study, and had almost normalized by day 6 post infection^44^. However, further studies are needed to evaluate the consequences of immune-mediated hepatitis.

In terms of clinical application the use of well tolerated adjuvants is of foremost importance. While a number of clinical trials indicated that CpG is sufficiently well tolerated, Poly(I:C) was shown to exhibit severe side effects in humans^45^. Of note, its synthetic derivative, PolyICLC, which has already been successfully used in DEC-205 targeting trials^46^,^47^,^48^ exhibits a better resistance to hydrolysis as well as a greater potency to induce IFNγ-secreting T cells^49^ than Poly(I:C) and, most importantly, was proven a safe adjuvant in healthy human volunteers^50^ and in cancer patients^51^,^52^. Additional studies are needed to clarify whether αDEC-205/OVA vaccination using PolyICLC/CpG as an adjuvant will be as efficient as (or even more efficient than) αDEC-205/OVA + Poly(I:C)/CpG vaccination in inducing antiviral immunity in the liver. Regarding the TLR2/6 agonist BPPcysMPEG which is known to activate cross-priming CD8α^+^ DCs in mice, so far no published data are available for clinical applications. It has recently been shown that human CD141^+^ DCs which are thought to represent the functional equivalent of mouse CD8α^+^ DCs do express TLR2 and 6^53^. Thus, an interaction of BPPcysOVAMPEG with DCs via the TLR2/6 receptors is likely to take place also in humans. Nevertheless, future *in vitro* studies are needed to clarify this issue in more detail.

We observed distinct patterns of CD8^+^ T cell interaction with virus infected and apoptotic hepatocytes, dependent on the vaccine formulation we used (Fig. 5C–E). While significantly fewer CD8^+^ T cells were in intimate contact with virus infected hepatocytes in the αDEC-205/OVA-immunized group, these CD8^+^ T cells appear to be more effective in inducing apoptosis which is in line with the higher expression of IFNγ as a marker for their cytotoxic potential. It is well established that CTLs eliminate infected hepatocytes after direct antigen recognition on target cells via perforin/granzyme or Fas-mediated killing. However, cytotoxicity in virus infected hepatocytes can also be exerted via the non-canonical CTL effector function where CTLs are stimulated by cross-presenting liver sinusoidal endothelial cells and secrete TNF after stimulation^54^. TNF induces cell death specifically in infected hepatocytes involving caspase-3 activation following TNFR stimulation. Of note, well in line with the observed elevated frequencies of IFNγ^+^CD44^+^CD8^+^ CTL in *AdOVA-GFP-luc* infected livers of αDEC-205/OVA compared to BPPcysOVAMPEG immunized mice, the overall IFNγ and as well TNF expression levels were by far higher in the αDEC-205/OVA treated group (Supplementary Fig. S3). IFNγ secreted by highly abundant IFNγ^+^CD44^+^CD8^+^ CTL may account for elevated TNF expression in hepatic macrophages in *AdOVA-GFP-luc* infected αDEC-205/OVA immunized mice. Alternatively, as shown before by Wohlleber *et al*.^54^, virus-specific CTLs induced during αDEC-205/OVA may be the source of TNF. While further investigation is needed to decipher the specific mechanisms underlying the elimination of virus infected hepatocytes in αDEC-205/OVA and BPPcysOVAMPEG vaccinated mice, we may speculate that immune surveillance in αDEC-205/OVA immunized mice in addition to cell-contact dependent CTL-mediated killing to a higher degree involves cytokine-mediated effector functions. At first line TNF may be the most important cytokine involved in elimination of virus infected hepatocytes as it can induce apoptosis in adenovirus infected hepatocytes. Although IFNγ has been shown to exert non-cytolytic antiviral effector function in HBV infected hepatocytes^55^, previous reports have shown that IFNγ ko mice behave exactly the same as wildtype mice with regards to antiviral immune response against adenovirus infected hepatocytes and following liver damage^28^,^54^. This does not exclude that high levels of IFNγ may enhance secondary antiviral effects for example by the upregulation of antigen-presentation by hepatocytes enhancing recognition of infected cells by CTLs.

The induction of antiviral immunity in the liver is *per se* a challenging issue. A hallmark of e.g. HCV persistence is the appearance of functionally impaired and exhausted T cells that are unable to secrete antiviral effector molecules, show impaired proliferation^29^,^56^,^57^ and dysregulate expression of activating/inhibitory receptors^58^,^59^,^60^. Next to this, in patients chronically infected with HCV the higher frequency of suppressive CD4^+^CD25^+^ T regulatory cells^61^ most likely contribute to T cell dysfunction. Since blocking of PD-1, CTLA-4 and Tim-3 hold some therapeutic promise for the functional recovery of exhausted T cells^59^,^60^,^62^, a combined strategy encompassing both antibody-mediated blocking of immunosuppressive pathways together with boosting antiviral immunity by means of DEC-205- or TLR-mediated antigen delivery to cross-presenting DCs could represent a promising scenario for future developments. This may also imply the use of DC targeting as a therapeutic vaccine, which has been shown to be clearly effective in several other studies^14^,^21^,^25^, and might also contribute to the treatment of patients with hepatic virus infection after liver transplantation to avoid reinfection.

## Methods

### Mice

Female C57BL/6 mice were obtained from Harlan Winkelmann (Borchen, Germany) and housed under specific pathogen-free conditions according to the national and institutional guidelines. All experiments were approved by the local government agency (Niedersächsisches Landesamt für Verbraucherschutz und Lebensmittelsicherheit; file number 33.12-42502-04-10/0108) and have been performed in accordance to these guidelines.

### Antibodies, antigens and cell lines

EndoGrade OVA was obtained from Hyglos (Germany). OVA peptides CD4_323–339_ (ISQAVHAAHAEINEAGR) and CD8_257–264_ (SIINFEKL) were synthesized at the Helmholtz Centre for Infection Research (Germany). The DEC-205 antibody was purified from NLDC-145^63^ hybridoma supernatant by affinity chromatography.

### Conjugation of OVA to αDEC-205

Chemical conjugation of purified αDEC-205 to OVA was performed as previously published^64^ with minor modifications. In brief, αDEC-205 was first activated by sulfo-SMCC (sulfosuccinimidyl 4-[N-maleimidomethyl] cyclohexane-1-carboxylate) (Thermo Fisher Scientific, USA), according to the manufacturer’s protocol, and in parallel the sulfhydryl-groups of the OVA protein were exposed by using 30 mM tris(2-carboxyethyl)phosphine hydrochloride (TCEP-HCl) (Thermo Fisher Scientific, USA) (1.5 hours at room temperature). Excess of TCEP and sulfo-SMCC in the respective samples was removed using Zeba Desalt Spin Columns, according to the manufacturer’s recommendations (Thermo Fisher Scientific, USA). The reduced OVA was immediately mixed with the activated antibody and incubated overnight at 4 °C. The resulting αDEC-205/OVA conjugate was concentrated and separated from unbound OVA using 150 K MWCO Pierce^®^ concentrators (Thermo Fisher Scientific, USA). Quantification of the OVA content within the αDEC-205/OVA conjugate was achieved by comparison of OVA signal intensities on Western blot with graded quantities of OVA on the same blot (Supplementary Fig. S4)^14^. Quantitative proportions of OVA peptide epitopes contained in the αDEC-205/OVA, BPPcysOVAMPEG and OVA + BPPcysMPEG vaccine formulations calculated on the basis of molar mass were determined as 1:7 (αDEC-205/OVA:BPPcysOVAMPEG), 1:12.5 (OVA + BPPcysMPEG:BPPcysOVAMPEG) and 1:1.7 (OVA + BPPcysMPEG:αDEC-205/OVA).

### TLR ligands

Poly(I:C) and CpG were purchased from Invivogen (Germany) and Eurofins MWG Operon (Germany), respectively. BPPcysMPEG (International Patent Classification: A61K47/48(2006.01), Pub. No.: WO/2007/059931) and BPPcysOVAMPEG (PCT/EP 09016050.8) were synthesized at the Helmholtz Centre for Infection Research (Germany).

### Immunization

Mice were subcutaneously immunized on days 0, 14 and 28 with either 30 μg αDEC-205/OVA, 30 μg αDEC-205, 10 μg BPPcysOVAMPEG^12^ or 7 μg OVA co-administered either with 50 μg Poly(I:C) and 50 μg CpG, as previously published^15^,^16^,^24^,^25^ or 5–10 μg BPPcysMPEG^39^ in a total volume of 50 μl PBS per animal.

### Determination of serum ALT

For ALT quantification, 75 μl peripheral blood were mixed with 25 μl 1% heparin (Ratiopharm, Germany), centrifuged (10,600× g, 10 minutes, room temperature) and 32 μl of the supernatant were used for detecting ALT activity using the scil Reflovet^®^ Plus reflection-photometer (scil animal care, Germany).

### Analysis of liver lymphocytes

Livers were perfused with ice-cold PBS and minced on ice followed by enzymatic digestion in IMDM (Gibco, Germany) containing 0.2 mg/ml collagenase D (Roche, Germany), 10 μg/ml DNase (Sigma, Germany) and 5% FCS for 30 minutes at 37 °C. After addition of EDTA (5 mM final concentration), cells were pelleted by centrifugation (15 minutes at 300× g) and liver lymphocytes were enriched by Percoll density gradient centrifugation. The cells were stimulated with a mixture of OVA protein and the OVA peptides CD4_323–339_ and CD8_257–264_ (20 μg/ml final concentration for all) for 24 hours at 37 °C. The cells were then stimulated by incubation with 0.01 μg/ml PMA (Sigma, Germany) and 1 μg/ml ionomycin (Sigma, Germany) for a total of 4 hours, 10 μg/ml Brefeldin A (Sigma, Germany) were added after 2 hours. For flow cytometric analysis, Fc-block was performed through incubation with anti-mouse CD16/CD36 antibody (2.4G2) followed by surface staining for mouse CD8 (53–6.7) and CD44 (IM7) (BD Biosciences, eBioscience). After fixation (2% paraformaldehyde/PBS v/v), cells were permeabilized using 0.1% Igepal^®^ CA-630/PBS (Sigma, Germany) and stained for intracellular IFNγ (XMG1.2). Data were acquired on an LSR Fortessa instrument (BD Biosciences) and further analysed using the FlowJo software (Tree Star, USA).

### Detection of antigen-specific serum IgG

To monitor the humoral immune response, 75 μl of blood were collected from the retro-orbital sinus and serum was obtained through incubation of the samples for 45 minutes at 37 °C, followed by 45 minutes incubation at 4 °C and subsequent centrifugation (10 minutes at 420× g). Sera were assayed for the presence of antigen-specific IgG and IgG subclasses (IgG_1_, IgG_2c_) by enzyme-linked immunosorbent assay (ELISA) using 96-well Nunc-Immuno MaxiSorp plates (Nunc, Germany) coated with 2 μg/ml OVA protein in 0.05 M carbonate buffer (pH 9.6). After overnight incubation at 4 °C, the plates were washed with PBS supplemented with 0.1% Tween 20 and blocked with 3% BSA in PBS for 1 hour at 37 °C. Serial 2-fold dilutions of sera in 3% BSA/PBS were added and plates were further incubated for 2 hours at 37 °C. After washing, antibody binding was detected using biotin-conjugated goat α-mouse IgG (Sigma, Germany), goat α-mouse IgG_1_ (Southern Biotech, USA) and goat α-mouse IgG_2c_ (Southern Biotech, USA) antibodies (1 hour, 37 °C), respectively, and streptavidin-HRPO (BD Biosciences, Germany) (30 minutes, 37 °C). ABTS in 0.1 M citrate-phosphate buffer (pH 4.35) containing 0.03% H_2_O_2_ was added to each well and the absorbance at 405 nm was recorded after 30 minutes of incubation. Endpoint titres were expressed as the reciprocal value of the last serum dilution which yielded an absorbance two times above the values of negative controls.

### Enzyme-linked immunosorbent spot (ELISPOT) assay

ELISPOT kits for the detection of murine IFNγ (eBioscience, Germany) and IL-4 (BD Biosciences, Germany) were used according to the manufacturer’s instructions. Spots were counted with an ELISPOT reader (C.T.L. Europe GmbH, Germany) and analysed using the ImmunoSpot image analyser software v3.2 (C.T.L. Europe GmbH, Germany). The results are presented as spot forming units per 10^6^ cells.

### *In vivo* cytotoxicity assay

Splenocytes were isolated from naive syngeneic donor mice and equal cell numbers were pulsed with 1 μg/ml SIINFEKL (OVA_257–264_) peptide for 30 minutes at room temperature or left untreated followed by labelling with 2.5 μM (CFSE^high^) or 0.25 μM (CFSE^low^) CFSE, respectively, and mixing in a one to one ratio. A total of 2 × 10^7^ target cells was intravenously (i.v.) injected into recipient mice. After one day, splenocytes were re-isolated and the ratio of CFSE^high^ to CFSE^low^ labelled cells was determined by flow cytometry using a FACS Canto instrument (BD Biosciences, Germany).

### Adenovirus challenge assay

On day 42 after the first immunization, mice were infected intravenously with 2 × 10^8^ plaque forming units (PFU) of a recombinant adenovirus expressing fusion proteins either of GFP and luciferase (*Ad-GFP-luc*) or GFP, luciferase and the OVA_aa257–264_ SIINFEKL (*AdOVA-GFP-luc*). Control animals received PBS and were either mock-infected (PBS) or infected with *AdOVA-GFP-luc*^28^. For luciferase activity assays, the lower liver lobe was separated into two parts, weighed and tissue fragments were homogenized in proportional volumes of reporter lysis buffer (Promega, Germany) using Lysing Matrix D (MP Biomedicals, Germany) and a FastPrep-24 instrument (3 × 10 sec, 5.5 m/sec). After centrifugation (3 minutes, 10,000× g, 4 °C), lysates were mixed with Luciferase Assay Reagent II (Promega, Germany) and measured in a luminometer (Berthold Technologies, Germany).

### MELC analyses

Liver tissues of αDEC-205/OVA + Poly(I:C)/CpG and BPPcysOVAMPEG immunized mice were snap frozen and embedded into O.C.T. (Sakura Finetek). Liver samples from αDEC-205/OVA and BPPcysOVAMPEG mice displaying comparable ALT levels were in each case analysed by the MELC robot in parallel. Cryosections of 10 μm thickness, which adhere to silan-coated cover slides, were prepared on Leica Cryostat CM3050, fixed with 2% paraformaldehyde (Santa Cruz) and permeabilized with 0.2% Triton-X-100 before blocking with 1% BSA/PBS (Sigma) for 1 h. Detailed information on image acquisition and analysis are described in the Supplementary material. Optimal antibody dilutions, incubation times, and positions within the MELC experiment for all antibodies used (see Supplementary Table S1) were validated systematically using conditions suitable to MELC^65^ (see Supplementary Figure S5).

### Histological analyses

The left upper liver lobe was fixed in 4% formaldehyde solution, followed by paraffin embedding, preparation of 2–4 μm sections and staining with haematoxylin and eosin (H&E). Histological evaluation was performed by an animal pathologist certified by the American College of Veterinary Pathologists in a blinded fashion.

### Statistical analyses

Results were statistically analysed by one-way Anova followed by the Dunnett’s test, two-way RM Anova, non-parametric Mann Whitney test or the paired, two-tailed *t*-test using the Graph Pad Prism 5 software (Graph Pad software, La Jolla). Data are presented as mean ± SEM or mean ± SD and a *p*-value below 0.05 was considered significant.

## Additional Information

**How to cite this article**: Volckmar, J. *et al*. Targeted antigen delivery to dendritic cells elicits robust antiviral T cell-mediated immunity in the liver. *Sci. Rep.* **7**, 43985; doi: 10.1038/srep43985 (2017).

**Publisher's note:** Springer Nature remains neutral with regard to jurisdictional claims in published maps and institutional affiliations.

## Supplementary Material

Supplementary Information
