# Supplementary material for: Targeted antigen delivery to dendritic cells elicits robust antiviral T cell-mediated immunity in the liver
Source: Sci Rep. 2017 Mar 7;7:43985. doi: 10.1038/srep43985 (PMC5339819; doi:10.1038/srep43985)
Supplement: Supplementary Information [file srep43985-s1.doc]

**Targeted antigen delivery to dendritic cells elicits robust antiviral T cell-mediated immunity in the liver**

Julia Volckmar^1^, Marcus Gereke^1^, Thomas Ebensen^2^, Peggy Riese^2^, Lars Philipsen^3^, Stefan Lienenklaus^4,§^, Dirk Wohlleber^5^, Robert Klopfleisch^6^, Sabine Stegemann-Koniszewski^1^, Andreas J. Müller^3^, Achim D. Gruber^5^, Percy Knolle^5,7^, Carlos A. Guzman^2^, Dunja Bruder^1*^

## Supplementary figures

Supplementary Figure S1: Comparative analysis of humoral and cellular immunity induced following peptide-targeting to DCs via BPPcysOVAMPEG versus non-targeting approaches using soluble protein and peptides adjuvanted with BPPcysMPEG.

Mice (n=3) were immunized on days 0, 14 and 28 with 10 µg BPPcysOVAMPEG, 5 µg BPPcysMPEG (BPP) alone or co-administered with 10 µg OVA protein (OVA + BPP) and 5 µg of both the CD4^+^ and CD8^+^ OVA peptides with (CD4^+^/CD8^+^ OVA peptides + BPP) and without (CD4^+^/CD8^+^ OVA peptides) adjuvant, respectively. PBS served as negative control. Analysis of OVA-specific serum (A) IgG, (B) IgG_1_ or (C) IgG_2c_ on day 42 after the first immunization by ELISA expressed as endpoint titres. Statistical significance was determined using the one-way Anova (mean ± SEM) followed by the Bonferroni test (*p<0.05). D-F) Analysis of IFNγ-producing splenocytes by ELISPOT on day 42 after the first immunization. Results are expressed as spot forming units per 10^6^ splenocytes detected following stimulation with (D) OVA protein, (E) OVA peptide CD4_323-339_ (ISQAVHAAHAEINEAGR) or (F) OVA peptide CD8_257-264_ (SIINFEKL). Bars represent the mean ± SEM (n=3, triplicates from pooled animals) of one experiment. Values obtained from non-stimulated cells were subtracted and results were statistically analysed using the one-way Anova followed by the Bonferroni test (*p<0.05; **p<0.01; ***p<0.0001). (G) Detection of IFNγ secretion by Flowcytomix array. Splenocytes of vaccinated mice were re-stimulated in quadruplicates with different concentrations of OVA protein (as indicated) for 96 h and IFNγ secretion was evaluated. Results are expressed in pg/ml.


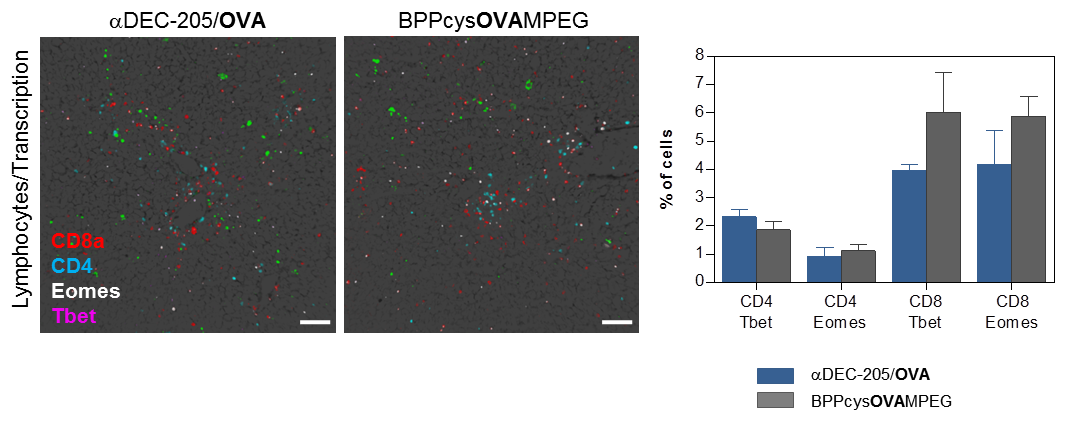


## Supplementary Figure S2: MELC analysis of liver tissue reveals similar distribution of CD4^+^ and CD8^+^ T cell subsets in αDEC-205/OVA and BPPcysOVAMPEG immunized and virus infected mice.

Mice (n=3-4) were immunized on days 0, 14 and 28 either with αDEC-205/OVA + 50 µg/50 µg Poly(I:C)/CpG or 10 µg BPPcysOVAMPEG and challenged with *AdOVA-GFP-luc*. 4 days after adenovirus infection, liver lobes were harvested for MELC analysis. Tissue sections of immunized and *AdOVA-GFP-luc* infected mice are shown (Scale bars = 100 µm). Red = CD8^+^ T cell; blue = CD4^+^ T cell; white = Eomes; magenta = Tbet. Graph: quantification of the transcription factor expression in CD4^+^ and CD8^+^ T cells as percentages of all detected cells.

## MELC analysis:

### Image acquisition

Liver tissue samples were placed on the stage of an inverted wide-field fluorescence microscope (Leica DMI6000 or Leica DM IRE2, 20x air lens NA 0.70). Fields of view were manually defined (two fields per condition), a corresponding transmitted light reference image and the XYZ-coordinates were stored. A fully automated cyclic robotic process starts with the detection of the signal of the GFP-tagged virus. These signals were removed by bleaching before the incubation of the first fluorescently labelled antibody (tag) started. Following a series of washing steps the fluorescence signals and a corresponding phase contrast image were acquired by a cooled charge-coupled device camera (Apogee KX4; Apogee Instruments, Roseville, CA, 1× binning results in images of 2048×2048 pixels; final pixel size 900×900nm2). By bleaching the fluorescent dye the signal of the given tag was removed before a post-bleaching fluorescence signals was recorded and the next incubation-imaging-bleaching-cycle started with the next tag. These cycles were processed until all tags were applied to the sample.

### Image analysis

Using the corresponding phase contrast images the series of fluorescence images produced by each tag were aligned pixel-wise. The automated algorithm reaches an alignment accuracy of 0.1 pixels. Illumination faults of the images were corrected using flat-field correction. Post-bleaching images were subtracted from the following fluorescence tag images. Finally, cases of section artefacts were excluded as invalid by a mask-setting process.

A cell mask as a dilation of 2 pixels around the nucleus of the cell was defined in order to determine intensity based analysis, spatial distribution or relation of the recorded marker signals. For each of these cells the mean fluorescent intensity and the smallest distance to two reference object masks were calculated. These masks were automatically created for the GFP and active caspase-3 marker signals to define regions which are positive for virus infected cells or cells which potentially undergo apoptosis. The resulting matrix of intensities and distances were exported into an FCS file and uploaded to the online cytometry analysis platform “cytobank.org” for multi-parametric analysis.

| **Antibody** | **Clone** | **Supplier** | **Catalog Number** | **Concentration** | **Label** |
| --- | --- | --- | --- | --- | --- |
| active caspase-3 | C92-605 | BD Bioscience | 560626 | 1:20 | Alexa 647 |
| CD8a | 53-6.7 | Biolegend | 100724 | 1:40 | Alexa 647 |
| CD11b | M1/70 | eBioscience | 53-0112-82 | 1:80 | Alexa 488 |
| CD45 | 30-F11 | BD Bioscience | 553080 | 1:240 | FITC |
| F4/80 | BM8 | eBioscience | 53-4801 | 1:240 | Alexa 488 |
| Eomes | Dan11mag | eBioscience | 12-4875-82 | 1:240 | PE |
| Tbet | 4B10 | Biolegend | 644804 | 1:40 | Alexa 647 |
| NK1.1 | PK136 | Biolegend | 108718 | 1:40 | Alexa 488 |
| CD3e | 17A2 | Biolegend | 100210 | 1:80 | Alexa 488 |
| CD4 | RM4-5 | BD Bioscience | 557667 | 1:40 | Alexa 488 |
| CD45R | RA3-6B1 | Biolegend | 103225 | 1:120 | Alexa 488 |
| Actin | C4 | BD Bioscience | 558623 | 1:80 | Alexa 488 |
| CD138 | REA104 | Miltenyi Biotec | 130-102-528 | 1:12 | APC |

## Supplementary Table S1: Antibodies used for MELC analysis.

**A**

| gene |  | forward primer | reverse primer |  |
| --- | --- | --- | --- | --- |
| ifng | 5´ | AGGAACTGGCAAAAGGATGGTGA | TGTTGCTGATGGCCTGATTGTCTT | 3´ |
| tnfa | 5´ | CAATGCACAGCCTTCCTCACAG | CCCGGCCTTCCAAATAAATACAT | 3´ |
| rps9 | 5´ | CTGGACGAGGGCAAGATGAAGC | TGACGTTGGCGGATGAGCACA | 3´ |

**B**

**C**

**C**

## Supplementary Figure S3: Quantitative real-time PCR (qRT-PCR) reveals enhanced transcriptional activation of IFNγ and TNFα genes in the liver of αDEC-205/OVA immunized and *AdOVA-GFP-luc* infected mice.

Mice were immunized on days 0, 14 and 28 with 30 µg αDEC-205/OVA + 50 µg/50 µg Poly(I:C)/CpG or 10 µg BPPcysOVAMPEG and challenged (i.v.) with 2x10^8^ PFU *AdOVA-GFP-luc* *(AdOVA)* or control virus *Ad-GFP-luc* (AdGFP). Four days later, mice were sacrificed and total RNA from one liver lobe (n=2-4) was isolated for qRT-PCR analysis using primer pairs specific for (A) *ifng, tnfa* and *rps9*. 1 μg total RNA was used for cDNA synthesis (Invitrogen) and relative qRT-PCR was performed on a LightCycler 480 II (Roche) using Maxima SYBR Green /Rox qPCR Master Mix (Thermo Fisher Scientific). Gene expression of (B) INFγ and (C) TNFα was normalized to the housekeeping gene *rps9* and fold changes were calculated using the ΔΔCp method with efficiency correction. Groups of *AdOVA* infected mice were compared by unpaired, two-sided *t*-test (*p=0.02; ***p=0.0002).


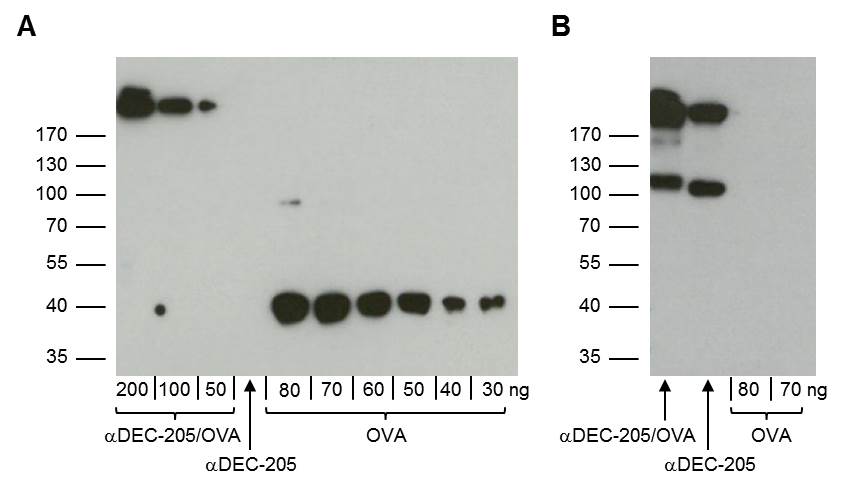


## Supplementary Figure S4: Characterization of αDEC-205/OVA conjugate.

The αDEC-205/OVA conjugate was verified by SDS-PAGE (non-reduced gel) and Western blotting utilizing (A) α-OVA (Acris Antibodies GmbH, Germany) and goat α-rabbit-(H+L) HRPO (Dianova GmbH, Germany) or (B) goat α-rat IgG (H+L)-HRPO (Dianova GmbH, Germany) in order to detect conjugated OVA or αDEC-205, respectively. To quantify the amount of OVA in the conjugate, different concentration of αDEC-205/OVA was subjected to Western blotting along with known quantities of OVA protein (as indicated). αDEC-205 served as control.


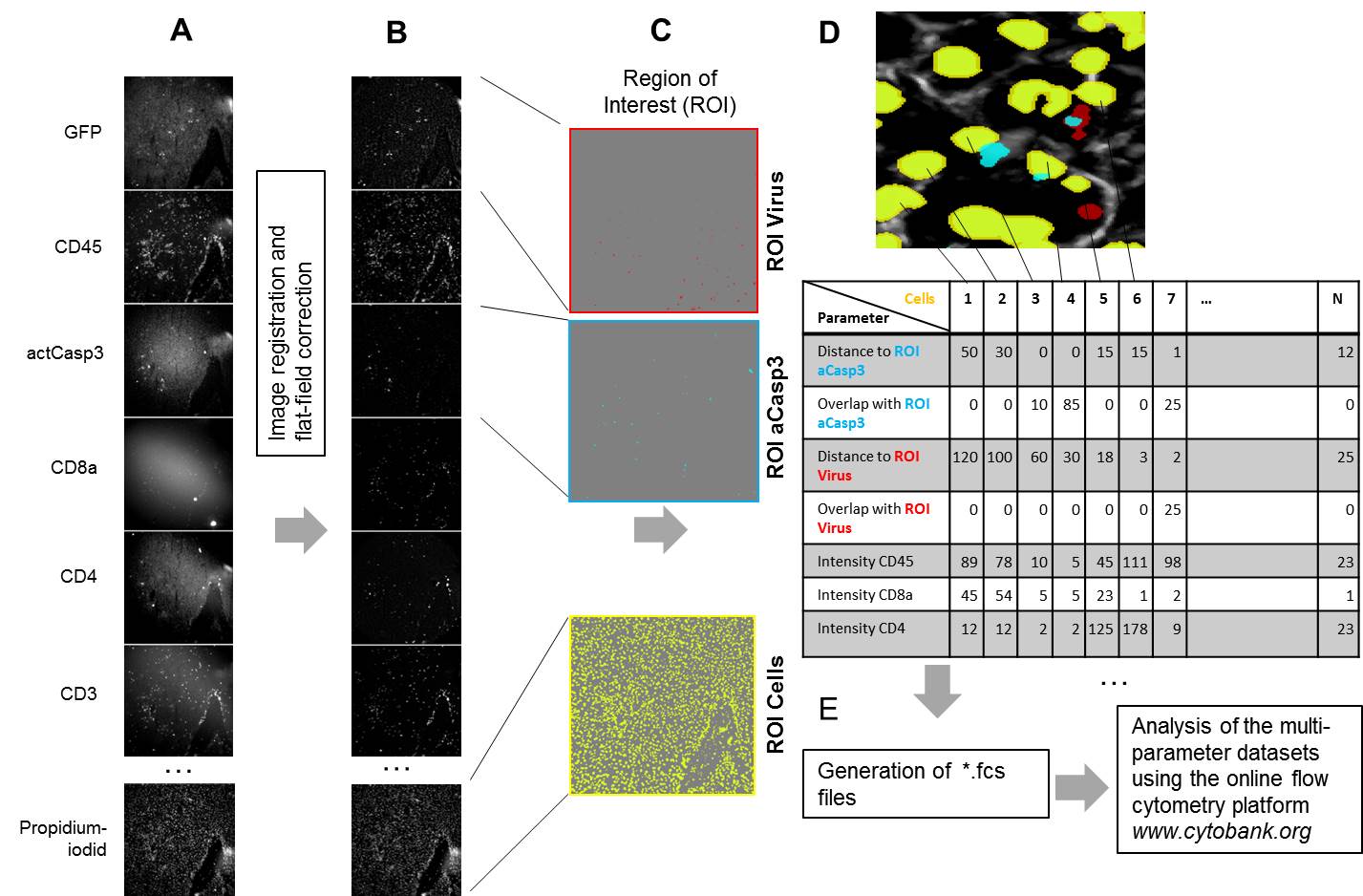


## Supplementary Figure S5: Image processing and data analysis.

(A) A series of raw fluorescence images acquired by the CCD camera of the MELC robot is shown. The corresponding phase contrast images (not shown) were used to align all images of the image series. Illumination fault due to the optics and the large CCD sensor were corrected using a flat-field image, which is selected from the series of post-bleach images. (B) Series of corrected fluorescence images of one single field of view is displayed. (C) Regions of interest are defined using an automated threshold based algorithm. Red indicates pixels positive for virus infected cells and cyan cells positive for active caspase-3 (actCasp3). The ROI Cells (yellow) represent all detected cells, which were derived from the mask of nuclei. (D) For each individual cell (yellow) the distances to the reference regions (e.g. cyan = active caspase-3; red = virus) or the pixel overlap can be determined. Arbitrary of these spatial data are shown in the first 4 rows of the table, whereas row 5 to end shows the mean fluorescent intensities of the markers for all cells. (E) Spatial data as well as the fluorescence data for all cells were stored into FCS 3.0 data files, uploaded to the online multi-parameter flow cytometry data analysis platform www.cytobank.org.
